# Organizing Heterogeneous Samples Using Community Detection of GIMME-Derived Resting State Functional Networks

**DOI:** 10.1371/journal.pone.0091322

**Published:** 2014-03-18

**Authors:** Kathleen M. Gates, Peter C. M. Molenaar, Swathi P. Iyer, Joel T. Nigg, Damien A. Fair

**Affiliations:** 1 Department of Psychology, University of North Carolina, Chapel Hill, North Carolina, United States of America; 2 Department of Human Development and Family Studies, The Pennsylvania State University, State College, Pennsylvania, United States of America; 3 Department of Behavioral Neuroscience, Oregon Health & Sciences University, Portland, Oregon, United States of America; 4 Department of Psychiatry, Oregon Health & Sciences University, Portland, Oregon, United States of America; 5 Advanced Imaging Research Center, Oregon Health & Sciences University, Portland, Oregon, United States of America; Duke-NUS Graduate Medical School, Singapore

## Abstract

Clinical investigations of many neuropsychiatric disorders rely on the assumption that diagnostic categories and typical control samples each have within-group homogeneity. However, research using human neuroimaging has revealed that much heterogeneity exists across individuals in both clinical and control samples. This reality necessitates that researchers identify and organize the potentially varied patterns of brain physiology. We introduce an analytical approach for arriving at subgroups of individuals based entirely on their brain physiology. The method begins with Group Iterative Multiple Model Estimation (GIMME) to assess individual directed functional connectivity maps. GIMME is one of the only methods to date that can recover both the direction and presence of directed functional connectivity maps in heterogeneous data, making it an ideal place to start since it addresses the problem of heterogeneity. Individuals are then grouped based on similarities in their connectivity patterns using a modularity approach for community detection. Monte Carlo simulations demonstrate that using GIMME in combination with the modularity algorithm works exceptionally well - on average over 97% of simulated individuals are placed in the accurate subgroup with no prior information on functional architecture or group identity. Having demonstrated reliability, we examine resting-state data of fronto-parietal regions drawn from a sample (N = 80) of typically developing and attention-deficit/hyperactivity disorder (ADHD) -diagnosed children. Here, we find 5 subgroups. Two subgroups were predominantly comprised of ADHD, suggesting that more than one biological marker exists that can be used to identify children with ADHD based from their brain physiology. Empirical evidence presented here supports notions that heterogeneity exists in brain physiology within ADHD and control samples. This type of information gained from the approach presented here can assist in better characterizing patients in terms of outcomes, optimal treatment strategies, potential gene-environment interactions, and the use of biological phenomenon to assist with mental health.

## Introduction

For many clinical disciplines disease status is initially identified from symptoms and verified via biological measures. For example, insomnia may be brought to a doctor's attention based on patient self-report, but the symptom can be attributed to diverse causes including lifestyle changes, hyperthyroidism, or Cushing's disease. Only through biological testing can the cause be identified and an appropriate treatment plan created. In contrast, the diagnosis and categorization of mental health disorders, such as attention-deficit/hyperactivity disorder (ADHD), typically relies solely on observable symptom clusters [1]. It is possible, if not likely [2], [3], that as with insomnia multiple mechanisms may lead to similar symptom clusters for a given mental disorder as defined by the Diagnostic and Statistical Manual of Mental Disorders (currently, DSM-5) or International Classification of Diseases (ICD). Clusters of symptoms that culminate to a diagnosis may relate to different neurobiological etiologies despite being under the same broad diagnostic category [4].

While it is largely agreed that the above possibility exists, studies examining the etiology of any given disorder typically ignore the potential heterogeneity that exists within current symptom-based classifications. In accordance with the current standard, analyses are conducted as though both the diagnostic group *and* the comparison control group represent two homogeneous populations. This approach makes two assumptions: (1) that the neurobiology for predefined diagnostic groups is distinct from those in another predefined group; and (2) that individuals are homogeneous within these predefined groups. A critical review of literature reveals that these assumptions are rarely met [5]–[7]. Moreover, assuming within-group homogeneity will likely cause misleading results at the aggregate level [8]–[10] in addition to missing important features for subpopulations within a given category.

This reality does not imply that current diagnostic categorization is completely arbitrary or not informative. However, it does present a significant barrier in the development of genetic and biological markers to assist in accurate diagnoses for a given disorder. Although small literatures have made attempts to parse fMRI data across individuals using various statistical methods (e.g., [11]), none have been sufficiently accepted to fall into wide use. Furthermore, efforts to subdivide based solely on biological features are still extremely rare (with notable exception using heart rate data [12]). The development of treatments, preventative, and intervention strategies for a given individual also suffer from this confound. Motivated by these issues, we present an approach for arriving at the potentially varied neurobiological etiologies related to a given mental health disorder, and show its utility in characterizing ADHD.

Specifically, we organize individuals into subgroups based on their brain connectivity maps. Brain connectivity maps have been used to identify systematic differences between subgroups and conditions in both clinical and non-clinical populations (e.g., [5], [13]). On these dynamic features of the individual, we conduct community detection to arrive at subgroups of individuals based entirely on their functional brain architecture. This approach will enable new insights into the number of biologically-based subgroups within any diagnostic category, including typically developing populations, by looking at the functional connectivity of regions.

We sought an approach that would be widely applicable, accessible, and useful. Since we wished to move away from classification of surface symptoms and towards classification based on neurobiological features of the brain, we required an algorithm for arriving at data-driven classification using no *a priori* information regarding category status or symptoms but rather uses features of brain functioning that are in line with current theories and approaches for understanding brain processes. Accordingly, several points of consideration helped guide the search for an optimal approach. For one, the method must be useful on data that comes from non-invasive techniques for understanding brain physiology. Additionally, since not all individuals with mental disorders are capable of doing difficult paradigms and experimental designs differ greatly across sites, the method should not require examining responses to experimental manipulation. Two, given that brain processes can best be thought of as coordinated activity of disparate regions across time [14], [15], we necessitated an approach for arriving at precise brain connectivity maps that quantifies relations among brain regions across time for each individual. This requires a state-of-the-art statistical method tailored to the data that detects signal from noise. Three, the algorithm must organize the individual-level models into subgroups based on brain connectivity estimates without prior classification information.

**Figure 1:**
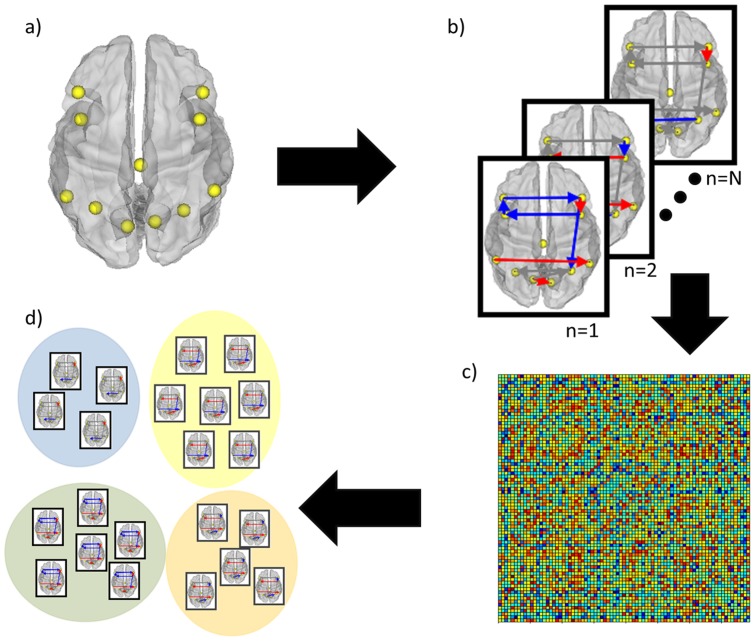
Schema of analytic process. Analysis were conducted using the following steps: a) obtain timeseries of functional MRI observations from regions of interest; b) arrive at directed functional connectivity maps for each individual using GIMME; c) correlate each individual's vector of connection weights with every other individual's vector for a similarity matrix; d) apply Newman's modularity maximization algorithm to similarity matrix to arrive at subgroups of individuals based on similar brain connectivity patterns.

Our method (see Figure 1) satisfies these requirements by combining: (1) functional MRI (fMRI) collected while participants are not engaged in a specific task (i.e. resting-state functional connectivity MRI – rs-fcMRI; [16]); (2) unified structural equation modeling (uSEM) [17], [18] conducted with Group Iterative Multiple Model Estimation (GIMME) [10] to ensure accurate individual level measurements of functional connectivity among brain regions; and (3) a widely used community detection algorithm [19], modularity, to arrive at data-driven subgroups of individuals based on brain processes rather than clusters of signs and symptoms. A benefit of rs-fcMRI is that it is developmentally and contextually more variable than brain structural measures, making it a highly attractive place to start towards the goal of identifying subgroups of individuals based on similar brain processes. Furthermore, it can be administered at across any age range, species, or cognitive ability. Prior rs-fcMRI analyses have revealed that there is ongoing information processing between spatially disparate regions of the brain even during rest, and differences in these observed processes relate to cognitive performance as well as psychiatric disorders. Connectivity maps conducted on rs-fcMRI data thus carry vast potential to advance understanding of normative and suboptimal brain processes. Taken together, our approach is a robust and easily applied method that utilizes functional connectivity networks to arrive at brain-based subgroups with no prior classification information.

## Methods

### GIMME

A necessary requirement for any project aiming to make inferences from directed functional connectivity maps is that they be reliable. Recent work demonstrated that most methods fail in their ability to recover both the presence and direction (i.e., which ROI statistically predicts the other ROI as opposed to bidirectional correlation between the two) of brain connections for individuals [20]. We utilize one of the only approaches to date that has been shown to reliably recover these parameters in heterogeneous and homogeneous samples of individuals: Group Iterative Multiple Model Estimation (GIMME; http://www.nitrc.org/projects/gimme/) [10]. GIMME first looks across individuals to detect signal from noise to arrive at a map that contains lagged and contemporaneous directed connections that exist for the majority (“group map”). In a second step GIMME identifies individual-level connections using the group-derived parameter patterns as a starting point. This has been shown to vastly improve reliability and precision of individual-level connections [10]. GIMME estimates the weight of these connections from within a unified Structural Equation Model (uSEM) [17] framework. Unified SEM contains both lagged and contemporaneous directed relations among regions, making it ideal for biological systems that likely contain these effects as detailed in [18].

### Subgrouping with community detection

For the final step, we utilize Newman's commonly used modularity algorithm [19] found in the Brain Connectivity Toolbox (http://www.brain-connectivity-toolbox.net) to arrive at data-driven subgroup classifications of individuals using solely the results from the directed functional connectivity analysis. Prior work in fMRI using this modularity algorithm required that the researcher set an arbitrary threshold, or cutoff point, for what constitutes similarity between any two given individuals. Researchers would then arrive at thresholds by looking for consistency in community detection results at multiple thresholds (e.g., [2], [21], [22]). Rather than rely on subjective cut-offs we utilized an entirely data-driven approach to thresholding guided by the same principals.

Modularity uses an input matrix which indicates the relatedness among N nodes, which in this case is n = 1…N individuals. To obtain this matrix, a first step in the present approach is to vectorize each individual's contemporaneous connection weights as estimated by GIMME. Recent work suggests that contemporaneous relationships best capture neuronal relations from BOLD data [20]. Including the lagged relations via uSEM ensures unbiased estimates of these contemporaneous relations [18]. Given the high degree of heterogeneity seen in the individual-level connections, including the individual-level paths may result in groups too small to offer any value over individual-level analysis. Thus we aim to subgroup individuals based on the similarities they share in their values in the sample-level paths. While only the sample-level connections are included to arrive at a correlation coefficient for each pair of individuals, it is necessary to estimate the individual-level connections when identifying the final models because these connections will ensure precise estimates of the connections we wish to use [23]. An added benefit is that sample-level connections will have normally distributed estimates across individuals, making it appropriate for the correlation analysis to follow.

The strength of linear dependence for the beta vector of each individual “i” with the beta vector of each other individual “j” is computed to produce a correlation matrix X. Following common practice in fMRI the weighted N by N matrix X is then binarized to create an A matrix where A_ij_ indicates if individual “i” is similar to individual “j” according to a given threshold *r*:
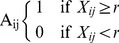
(1)which can then be used in Newman's modularity maximization algorithm; full equations for the method are explained in [19].

Deciding on the value for *r*, the threshold for which individuals are considered similar constitutes a pivotal decision in the algorithm. Researchers commonly select thresholds (or solutions) that provide the highest modularity index. Two criteria drive our threshold selection algorithm. One, the classification must be reliable across multiple runs. The modularity index can demonstrate considerable variability across iterations because it is sensitive to starting conditions that change randomly across runs. Researchers do not always consider this specific problem. Two, each individual must be reachable (i.e. have a connected path from one individual to another) by most other individuals at the given threshold [15]. Here, reachability is defined as the average number of individuals that each person can reach.

**Figure 2:**
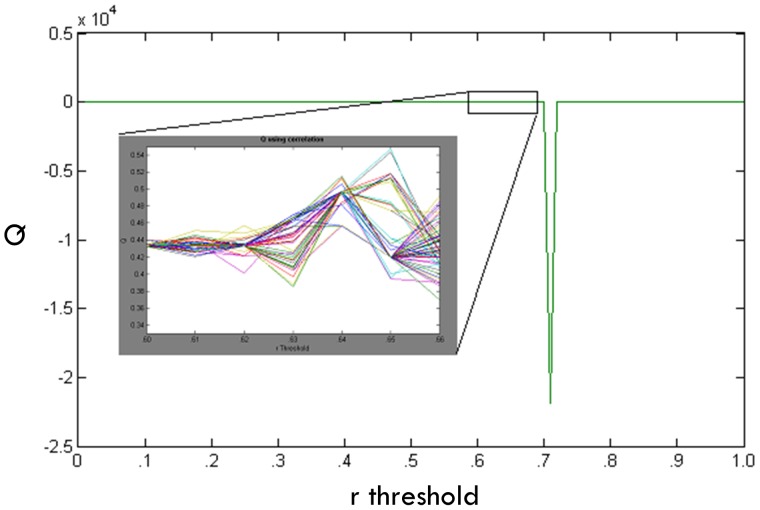
Instability in Q index. This is taken across 100 iterations at each threshold from 0.0 to 1.0 at .01 increments.

**Figure 3:**
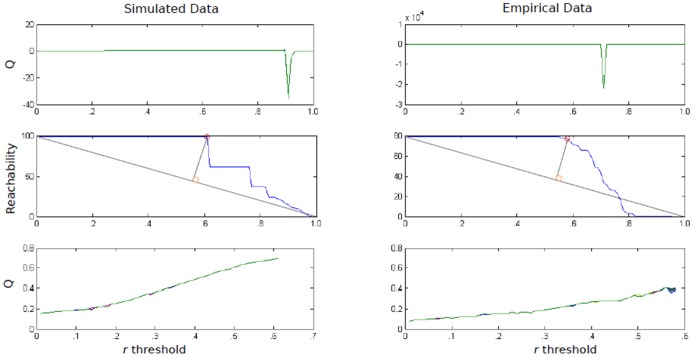
Q index and reachability across thresholds. Q values across all thresholds (top panel); average number of individuals each individual can reach at each threshold (i.e., reachability) and visual depiction (grey, dotted lines) of approach for defining the point at which reachability begins to decrease (middle panel); Q values (bottom panel) up until drop in reachability (denoted with a red circle in the middle panel). The optimal r thresholds with maximum stability, Q index value, and reachability criteria were .60 and .56 for the simulated data example presented here (1 of 100 runs) and empirical data, respectively.

The modularity algorithm [19] is conducted at each threshold 100 times. To investigate the first criteria, the stability of Q across these 100 community detection attempts is examined. Figure 2 illustrates the instability seen in the Q value. This instability corresponds to different subgroup denotations, such that choosing a subgroup assignment for one run might differ from a second run at the same threshold. As depicted in Figure 2 and the top panel of Figure 3, the Q index becomes unstable at certain thresholds. This is a due to the program being sensitive to starting conditions, which change randomly across each run. An optimal *r* threshold would provide community solutions that are stable across each run, indicating that the solution is robust to different starting points.

Our next criterion is the reachability index. For each threshold we see that the reachability drops prior to the Q index reaching maximum instability (Figure 3, middle panel). This supports our decision to choose a threshold that occurs prior to the drop off in reachability as this relates to community quality. The middle panel of Figure 3 depicts the process for finding the drop in reachability. A line is generated from the first to last point of the average reachability across *r* thresholds. Next, we find the greatest distance from this line to the reachability values using perpendicular angles to determine the point at which reachability drops.

The bottom panel of Figure 3 shows the Q estimates for each threshold (conducted 100 times) up until the point where reachability drops. Please note that even at low values some thresholds are unstable, as indicated by fluctuations in the Q, whereas others reliably obtain the same Q across 100 attempts. Simulated and empirical data reveal that when Q is stable for this specific binary modularity algorithm, the subgroups are also stable (see Results below). The standard deviation of Q across 100 estimates is used to quantify stability. We select the threshold for which the Q index has maximum stability; in the event of a tie, the highest *r* with the highest Q is selected. The correlation threshold *r* is set and the above A matrix is created accordingly and used in the binary modularity algorithm [19].

### Verifying the robustness of community detection solution

We followed the analytic plan outlined by Kerrer and colleagues [24] for testing the robustness of community structures in our simulated and empirical data. We quantified the difference between each run using the variation of information (VI) criterion developed by Meilă [25]. We use the VI index here to look at the robustness of community structures across varying levels of perturbations. This helps identify if the community structure is dependent on a small proportion of the data points and thus easily attributed to chance. First, we arrived at a final solution for each of the simulated and empirical data examples as outlined above. Here, “network” refers to the binary matrix of similarity at the chosen *r* threshold. Second, we randomly perturb the networks in a manner that retains the degree, or the number of edges, for each individual (i.e., vertex) by removing edges in the original network and placing it between two other individuals in the same network [24]. We conducted perturbations across probability levels which ranged from none (0) to completely random (1) at .025 increments. Each network (i.e., the 100 simulated data sets and the empirical data results) were randomly perturbed at each probability level 100 times. Third, we conducted community detection algorithm with data-driven thresholding for each of the perturbed networks to arrive at community structures. Finally, we took the community structures from the perturbed networks and measured the variation of information between these community assignments and the original one.

### Comparing connectivity maps between subgroups

Variation in the connection weights of specific connections between two given ROIs has long been a focal point of fMRI research [17]. Having arrived at data-driven subgroups, group-level weights are compared between the subgroups to see how each subgroup differs from the average of the other subgroups. ANOVA with false discover rate (FDR) correction, at alpha of 0.05, was used to look at differences in beta weights for group-level connections [26]. As a comparison to the typical method for examining ADHD brain connectivity patterns, we also compare the connection weights between the control and ADHD groups using the diagnostic categorization.

## Data

### Simulated data

**Figure 4:**
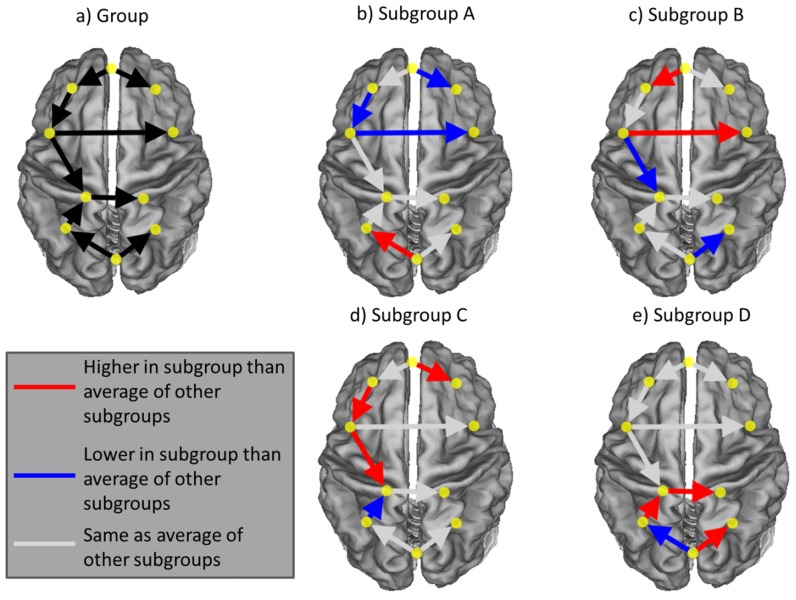
Patterns of effects used to simulate data and results.

Simulated time series of fMRI data for 10 brain regions for 100 individuals were used to demonstrate feasibility and reliability of our approach following the approach outlined in [10]. For the present simulation, each individual has a time series of length 200 scans (at 2 TRs). All ROIs for all individuals have autoregressive effects of weight 0.60. In addition to these and as depicted in Figure 4a, 9 contemporaneous paths exist for all individuals comprising this sample. These paths are generated to have a weight (technically “beta”) of 0.50 unless specified otherwise. For each subgroup, 3 of the 9 group-level beta estimates differ from .5 by ±.2 (refer to Figures 4b–e for specifics). Additionally, in each subgroup there exists one unique connection that randomly occurs across individuals comprising that subgroup using a binomial distribution with a probability of 0.5. This last level of heterogeneity adds in some individual-level variation within the group. The degree of similarity to expect as well as the variability in connection weights has been informed by previous research that used individual-level connectivity maps to examine a priori defined subgroups (e.g., [5])

### Empirical data set

We demonstrate the utility of this approach in a sample of 80 children (29 females and 51 male) aged 7–12 years old, of which 40% met DSM-IV and DSM-5 diagnostic criteria for Attention-Deficit/Hyperactivity Disorder (ADHD) by a multi-method, multi-informant, best-estimate procedure that we have described in detail elsewhere [27], [28] and summarized below. Written informed consent was obtained from parents and written informed assent from all child participants in accordance with the guidelines of the Oregon Health and Sciences University (OHSU) Research Integrity Office (IRB). The OHSU IRB approved this study.

Psychiatric diagnoses were based on multi-method, multi-informant research evaluations by our team with 1) *Kiddie Schedule for Affective Disorders and Schizophrenia* - KSADS-I [29], [30] administered to a parent, 2) parent and teacher (short form) *Conners' Rating Scale-3^rd^ Edition* [31] and Strengths and Difficulties Questionnaire (SDQ), (3) IQ and academic screening, (4) behavioral observations by a clinical interviewer, and then 5) a best-estimate clinical review by a board certified child psychiatrist and licensed clinical neuropsychologist. They independently assigned all appropriate diagnoses, with adequate agreement (kappa>.75 for all disorders with base rate >5% in a larger sample of several hundred children they have reviewed) and kappa >.80 for ADHD. They conferenced any disagreements and readily reached agreement or else the case was excluded. Estimates of intelligence were evaluated with a three-subtest short form (Block Design, Vocabulary, and Information) of the *Wechsler Intelligence Scale for Children, Fourth Edition* (WISC-IV) [32]. Demographic details including diagnostic status and IQ is provided in the Table 1.

**Table 1:** Demographics of empirical sample.

	ADHD	TDC	Statistic
**Mean Age in Years**	9.68 (1.52)	9.13 (1.20)	t = 1.83, df = 78, p = .07
**% Male**	78%	46%	χ^2^ = 4.77, df = 1, p = .03
**IQ**	110.81 (15.11)	116.92 (13.75)	t = 1.76, df = 70, p = .08

Standard deviation in parentheses. Eight values for IQ are missing (5 ADHD, 3 TDC).

Children were excluded if they did not meet DSM-IV criteria for ADHD or criteria for typically developing control. Children were also excluded for an IQ<75, or if a history of neurological illness, chronic medical problems, sensorimotor handicap, autistic disorder, mental retardation, or significant head trauma (with loss of consciousness) was identified by parent report. Children were also excluded if they had evidence of psychotic disorder or bipolar disorder on the structured parent psychiatric interview or were in a current major depressive episode. Children prescribed short-acting stimulant medications were scanned after a minimum washout of five half-lives (i.e., 24–48 hours depending on the preparation); all other psychoactive medications were a rule-out. Typically developing control children (TDC) were excluded for presence of conduct disorder, major depressive disorder, or history of psychotic disorder, as well as for presence of ADHD. All children were right handed.

## Data Acquisition and Processing

Participants were scanned using a 3.0 Tesla Siemens Magnetom Tim Trio scanner (Siemens, Erlangen, Germany) with a twelve-channel head-coil at the OHSU Advanced Imaging Research Center. One high-resolution T1-weighted MPRAGE sequence lasting 9 minutes and 14 seconds (TR = 2300 ms, TE = 3.58 ms, orientation = sagittal, 256×256 matrix, resolution = 1^3^ mm) was collected. Blood-oxygen level dependent (BOLD)-weighted functional imaging data were collected in an oblique plane (parallel to the ACPC) using T2*-weighted echo-planar imaging (TR = 2500 ms, TE = 30 ms, flip angle = 90°, FOV = 240 mm, 36 slices covering the whole brain, slice thickness = 3.8 mm, in-plane resolution = 3.8×3.8 mm). Steady state magnetization was assumed after 5 frames (∼10 s). Three runs of 3.5 minutes each were obtained. During rest periods subjects were instructed to stay still, and fixate on a standard fixation-cross in the center of the display.

All functional images were preprocessed in the same manner to reduce artifacts. These steps included: (i) removal of a central spike caused by MR signal offset, (ii) correction of odd vs. even slice intensity differences attributable to interleaved acquisition without gaps, (iii) correction for head movement within and across runs, and (iv) within-run intensity normalization to a whole brain mode value of 1,000. Atlas transformation of the functional data was computed for each individual via the MPRAGE scan. Each run then was resampled in atlas space [33] on an isotropic 3 mm grid, combining movement correction and atlas transformation in one interpolation [34]. All subsequent operations were performed on the atlas-transformed volumetric time series.

Functional connectivity preprocessing followed prior methods [35]–[37]. These steps included: (i) a temporal band-pass filter (0.009 Hz<f<0.08 Hz) and spatial smoothing (6 mm full width at half maximum), (ii) regression of the whole brain signal averaged over the whole brain, (iii) regression of ventricular signal averaged from ventricular region of interest (ROI), and (iv) regression of white matter signal averaged from white matter ROI. Regression of first order derivative terms for the whole brain, ventricular, and white matter signals were also included in the correlation preprocessing. These preprocessing steps are thought to reduce spurious variance unlikely to reflect neuronal activity [38].

Subjects underwent several rigorous steps to correct for head motion during scanning. At the first level of correction (i.e., traditional motion correction), motion was measured relative to a reference frame (in this case, the middle frame of a BOLD run) and quantified using an analysis of head position based on rigid body translation and rotation. This procedure results in the rigid body transform defined by six motion parameters (3 translation, 3 rotation) typically generated by most functional MRI software tools. These 6 parameters were used as regressors in preprocessing to remove potential motion related artifact. In addition, in an effort to remove participants with egregious motion, we began our analysis by filtering those subjects with high movement runs based on root mean square (RMS). The data derived from the 6 motion parameters needed to realign head movement on a frame-by-frame basis were calculated as RMS values for translation and rotation in the x, y, and z planes in millimeters. Total RMS values were calculated on a run-by-run basis for each participant. Participant's BOLD runs with movement exceeding 1.5 mm RMS were removed. Last, frame-to-frame displacement (FD) was calculated for every time point. FD was calculated as a scalar quantity using a formula that sums the values for framewise displacement in the six rigid body parameters (FDi = |Δdix|+|Δdiy|+|Δdiz|+|Δαi|+|Δβi|+|Δγi|, where Δdix = d(i−1)x −dix, and similarly for the other five rigid body parameters) [21]. At each time point, if the FD was greater than 0.2 mm, the frame was excluded from the subject's time series by a placeholder row of missing values so the temporal ordering of scans was retained for uSEM analysis.

**Figure 5:**
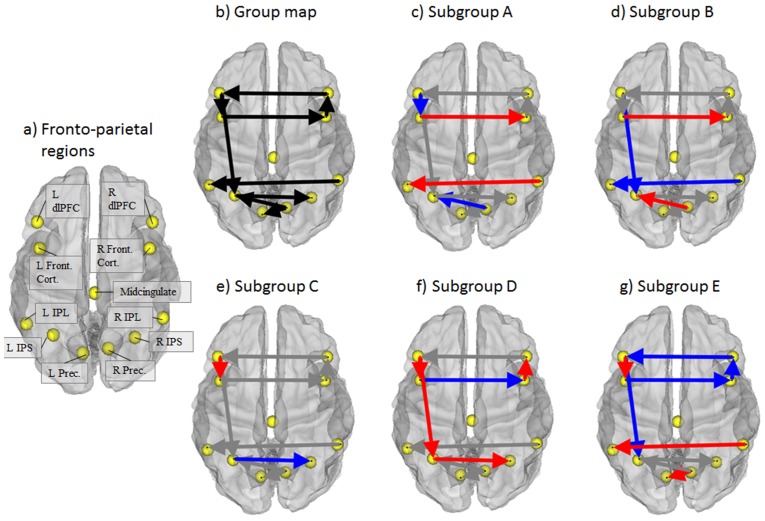
Regions and results from empirical sample. Red lines indicate the subgroup had higher connection values than the average of the other subgroups; blue lines indicate the subgroup had lower connection values than the average of the other subgroups; gray paths indicate the connection values were similar to the average of other subgroups. Abbreviations: “dlPFC” = dorsolateral prefrontal cortex; “FC” = frontal cortex; “IPS” = intraperietal sulcus; “IPL” = inferior parietal lobule; “R” preceding these ROI names and abbreviations denotes right and “L” denotes left.

We selected 11 regions of interest based on prior work by Dosenbach and colleagues [22], [39], [40]. These regions termed the fronto-parietal network (see Figure 5a) were selected based on their implied role in adaptive task level control that surfaces in resting state studies [40], [41] and atypical nature in studies of ADHD [42], [43]. Table 2 lists the 11 ROIs and coordinates. Time series were computed for each of the cortical regions by averaging the signal intensity across all voxels within a 10 mm sphere for each time point.

**Table 2:** Coordinate locations of regions in Talaraich space.

Region	X	Y	Z
L dlPFC	−43	22	34
R dlPFC	43	22	34
L FC	−41	3	36
R FC	41	3	36
midcingulate	0	−29	30
L IPL	−51	−51	36
R IPL	51	−47	42
L IPS	−31	−59	42
R IPS	30	−61	39
L precuneus	−9	−72	37
R precuneus	10	−69	39

“dlPFC” = dorsolateral prefrontal cortex; “FC” = frontal cortex; “IPS” = intraperietal sulcus; “IPL” = inferior parietal lobule; “R” preceding these ROI names and abbreviations denotes right and “L” denotes left.

## Results

### Simulated data

For the simulated data, GIMME was able to recover all of the connections which existed at the group level for each of the 100 sets of data (see Figure 4a). After considering individual-level connections (which varied systematically across subgroups), on average 99.73% (std. = 0.09%, range = 95–100% across the 100 data sets) of the connections across all individuals were recovered accurately (i.e., both the presence and direction were correct). The community detection algorithm described above then identified the subgroups excellently: 97.23% (std. = 1.87%, range = 92–100%) of the individuals within each data set were placed in subgroups with those who shared their simulated brain map pattern. ANOVA results consistently revealed significant differences in connection weights across the subgroups that corresponded with the patterns used to create the data that was consistent across the 100 simulated data sets (Figure 4b–e). This, taken together with the reliable recovery of subgroup classification, verifies that the present approach appropriately groups individuals who are indeed similar.

**Figure 6:**
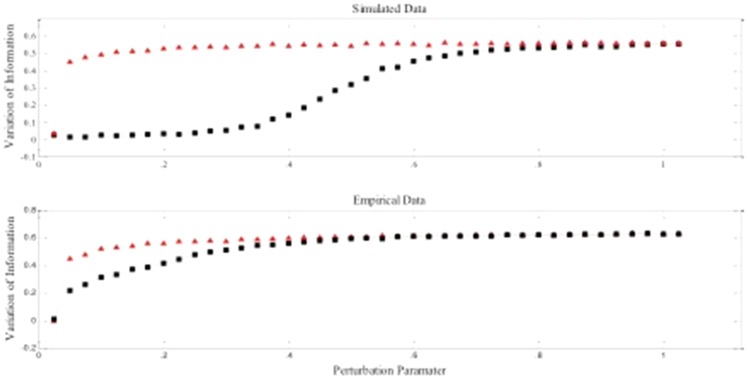
Variation of information (VI) in simulated and empirical data across varying degrees of perturbation. Red triangles indicate VI values obtained from random perturbations; black squares correspond to VI values obtained on the original matrices.

As an added check, we tested the modularity approach on randomly generated graphs to ensure robustness of our solution as described in the methods [24]. The subgroups were consistently recovered in the simulated data and were robust to minor perturbations. Figure 6a displays the results from a representative simulated data set (number 11 of 100). The simulated data results adhere to the ideal pattern described by Karrer and colleagues. The community assignments remain similar for the simulated individuals until a perturbation level of about .5 (i.e., random assignment of 50% of the edges), at which point 20% of the vertices are assigned different communities than the original and we see this variation of information increasing steadily past this point. The randomly generated graphs, by contrast, experience a sharp increase in variation of information at the slightest perturbation. Indeed, over 40% of the vertices have different community assignments after perturbing about two percent of the edges.

### Empirical data

The connectivity map depicted in Figure 5b provides the connectivity pattern of the fronto-parietal region set common for the majority of individuals in the whole sample (i.e. connections found to be significant for the majority, or the “group map”). In addition, a number of connections surfaced at the individual level (not pictured). Every individual had at least one connection in addition to those found at the group-level, and every potential connection between regions was found for at least one person. Hence there appears to be some connections that exist for the majority of individuals while there also exists a large degree of heterogeneity in the structure of connections as evidenced by the extra connections uncovered at the individual level. This finding highlights the need to use a method such as GIMME which is geared towards detecting signal from noise to reliably arrive at the presence of individual-level connections which in turn improve upon the precision of connection weight estimates.

We first compared path weights by considering the ADHD and Typically Developing samples to be two homogenous groups, akin to how current research projects are typically conducted. That is, we placed and analyzed the individuals into groups based on their diagnostic status. Here, our search is limited to differences between these two diagnostically defined groups. Tests for significant differences between ADHD and TDC on the path weights revealed no significant differences. Hence it appears the within-group heterogeneity washed out effects of interest that we were able to isolate by enabling for heterogeneity within the diagnostic categories.

**Figure 7:**
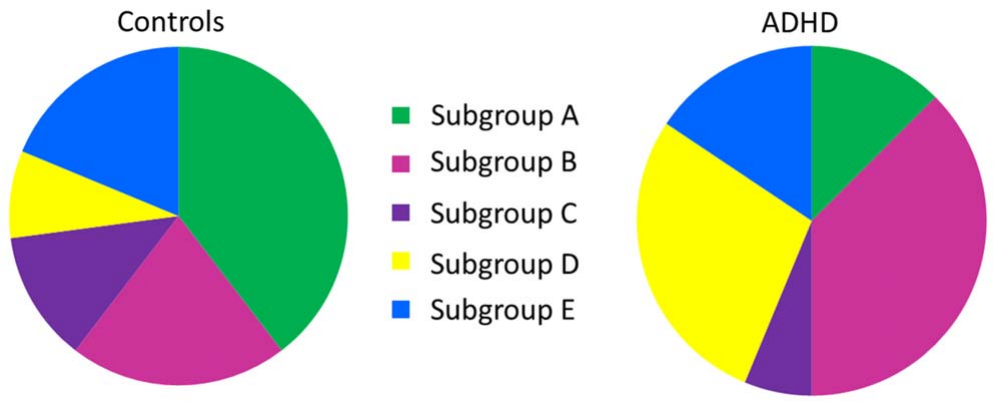
Subgroup make-up: Controls and ADHD.

We then applied the community detection algorithm to group individuals based on their brain patterns of connectivity as opposed to their diagnostic status. The community detection approach arrived at 5 subgroups of individuals. Regarding the robustness of the solution as quantified using VI, the curves for the empirical and random data remain distinct and thus the network of individuals can be said to show community structure (see Figure 6b; [24]). Since there are a higher proportion of males than females diagnosed with ADHD, we first investigated if males and females were evenly distributed across the subgroups. The proportions of males and females in each of the subgroups were not different than what one would expect by chance (χ^2^ = 3.65, df = 4, p = .456). ADHD and TDC participants, however, were placed in different subgroups at levels greater than chance (χ^2^ = 12.32, df = 4; p = .015; Figure 7). Of note, 38% of the controls are in subgroup A (see Table 3 for results). Subgroups B and D contain a high percentage of the ADHD children, who make up a large part of these subgroups. The smaller subgroups C and E are disproportionately control, but contain relatively small percentages of the control sample. Taken together, subgroups A, C, and E are subgroups with low likelihood for ADHD and as such have an organization of brain physiology that we term “protective.” That is, individuals with brain connectivity maps similar to those found in these groups will likely not have ADHD. Subgroups B and D appear to have brain organizations that place them at higher likelihood for having ADHD. These findings demonstrate that heterogeneity exists within both control and ADHD populations, and that the potential differences between the two categories are washed out when assuming within-group homogeneity.

**Table 3:** Group affiliation by ADHD diagnostic category.

	Subgroup				
	A	B	C	D	E
**Control (N = 48)**	18	10	7	4	9
*% of Control*	38%	21%	15%	8%	19%
*% within Subgroup*	82%	45%	77%	31%	64%
**ADHD (N = 32)**	4	12	2	9	5
*% of ADHD*	13%	38%	6%	28%	16%
*% within Subgroup*	18%	55%	22%	69%	36%

Numerous differences in the connection weights existed across the groups (Figures 5c–g). For instance, subgroup A (protective) was the only subgroup to have stronger interhemispheric connections than the average of the other subgroups in both the anterior and posterior regions. In subgroup B (risk), by comparison, the statistical prediction of the left inferior parietal lobule (L IPL) from the right inferior parietal lobule (R IPL) was weaker, but like subgroup A had strong directed influence of the left frontal cortex (L FC) on the R FC. Importantly, subgroup B had lower directed connectivity strength from the left dorsolateral prefrontal cortex (L dlPFC) to the left intraparietal sulcus (L IPS), which is consistent with previous research on deficits in attention [44]. However for this connection, subgroup D (also a risk group for ADHD) evidenced a stronger directed connection, suggesting that more than one pathway for attention deficits exists. Indeed, subgroup D (risk) appears to have decreased connectivity from the L FC to the R FC when compared to the average of the other groups, indicating a potential biological marker for ADHD in addition to the L dlPFC and L IPS deficit found in subgroup B and previous research. Subgroup E (protective) had decreased connectivity on a number of connections predominantly associated with the frontal regions. Taken together, there appears to be different physiological markers within the ADHD diagnostic category as well as different profiles within the control sample.

## Discussion

The current analysis presents a beginning effort at establishing empirically driven, brain-based subtypes in mental disorders. We combined and extended state-of-the-art methods to arrive at a dynamic community detection approach for characterizing differences in brain physiology, tested it using Monte Carlo simulations, and validated the approach with empirical data. Our approach differs from current implementations in at least two meaningful ways. For one, similarities among individuals are assessed using the entire pattern of connections as opposed to alternative methods that simply take one aspect, such as one connection weight or statistical parametric maps. In this way our approach is directly in line with the current understanding of brain functioning as best understood as the connectivity between disparate regions as opposed to isolated areas. Two, no assumptions are made regarding the classification of individuals. Traditional methods, such as machine learning or discriminant analysis, require *a priori* distinction of subgroups based on the construct of interest (e.g., gender, age, clinical category, performance). Humans are multidimensional and can be categorized on various axes [2], [45]. For this reason, a complementary way to look at group differences is presented here. The present approach organizes individuals based on their functional brain physiology, from which researchers can then examine how a given group relates to demographic, clinical, and performance characteristics.

The feasibility of using GIMME and community detection to arrive at data-driven subgroups of individuals based on brain physiology was demonstrated via our simulations. As seen previously [10], GIMME reliably recovered the presence and true direction of connections at the group and individual levels and is one of the few approaches that can do so [46]. Next, a widely used algorithm for arriving at subgroups, the modularity approach [19], was used for subgroup classification. This procedure was modified to arrive at the optimal solution in an entirely data-driven fashion. Subgroup designations were accurately made using the GIMME individual-level connectivity map estimates.

The empirical data example offered concrete evidence for theories that have hitherto been untested. That is, within diagnostic categories there exists heterogeneity in brain physiologies. In particular, researchers have made strong assertions that clinical diagnoses may result from multiple etiologies [3], [4]. This possibility has been noted in ADHD as well as Autism Spectrum Disorder [3], [47]. Most evidence for this phenomenon has been from neuropsychological data and behavior or symptom report (e.g., [2]), with recent evidence suggesting heterogeneity in the biological components as well [48]. In this sample, ADHD appears to have at least two main biological manifestations related to the fronto-parietal regions that differentiated individuals within this diagnostic category. In line with previous research [44], one of the ADHD-dominant subgroups did have weaker dorsolateral prefrontal cortex connection with the inferior parietal sulcus. However, the other ADHD-dominant subgroup had increased connectivity between these two regions but had decreased connectivity between the right and left frontal cortex. Hence grouping all ADHD individuals together may not capture all of the informative characteristics of the disorder and in this sample, washed out meaningful differences.

Although the majority of ADHD-diagnosed children fell into two subgroups, about a third were spread across the three subgroups comprised of predominantly typically developing control. This further supports the notion that many mechanisms exist by which children may meet criteria for ADHD [2]. If these results were taken at face value, we might propose for example that the current diagnostic criteria capture a group with a developmental brain trajectory that A) is at risk, and B) is sensitive to our functional MR measurements. We also would propose that another group with ADHD has either typical brain development or a pathology that likes outside of the brain systems examined here or for which our MR measurements are not sensitive to. Nonetheless, the work supports the potential of a biologically based nosology in the future.

A final important finding was that children identified as typically developing controls were found in every subgroup. Indeed, some individuals had biological markers that are similar to those in a clinical category yet did not meet the level for a clinical diagnosis. Investigating contextual indices that may protect those at biological risk for developing a disorder provides another utility for data-driven subgroup classifications. Thus it is interesting to consider that the brain findings here may reflect liability for ADHD in a biologically at risk subgroup, rather than ADHD per se—the controls in these groups may represent individuals at risk for ADHD who had sufficient protective factors in their development (or their genome) to avoid exhibiting the syndrome. It will be interesting to follow these groups over time to evaluate whether this type of hypothesis holds true over time.

Importantly, the fact that typically developing controls may be heterogeneous in functional brain architecture, or brain “profiles”, is not a new concept either. A long line of research in the social sciences has urged researchers to examine processes at the individual level rather than aggregate as is typically done in cross-sectional studies [45], [49]–[51]. Support for this notion is found in fMRI data, where individual differences in brain patterns relating to indices such as performance [17], [52] and gender [13] have been found in normative samples. The approach presented here provides a bridge between group-level aggregation and individual-level analysis. *A priori* grouping based solely on diagnostic category would miss the heterogeneity that exists within a diagnostic category, whereas conducting individual-level analysis would result in unique maps from which no meaningful inferences could be made.

Amidst the heterogeneity within diagnostic categories, subgroups emerged that were predominantly clinical or control, supporting some degree of biological validity to the existing nosology. Although both ADHD and control individuals surfaced in each of the five brain-based groups, the composition of each subgroup contained a disproportionate number of the diagnostic categories. Hence, our method can be used to identify disease-associated brain physiologies much like the current approach of comparing predefined a diagnostic group to a control group. Varied interhemispheric and posterior-anterior functional connections differentiate predominantly ADHD-comprised subgroups from predominantly control subgroups in our study. Data in the ADHD literature examining functional differences in the diagnostic subtypes (i.e. Combined type, and Inattentive type) lend support for this particular finding [2]. Most important, the approach presented here can help researchers better understand between-group differences alongside within-group heterogeneity.

The present work builds from prior subgrouping algorithms that have demonstrated success. However, community detection does not come without shortcomings. One, the popularity of community detection algorithms in fMRI research has grown substantially in a short time. Along with this growth there has been an influx of algorithms that seek to improve upon the traditionally used ones (e.g., [53]). The proliferation of community detection programs has occurred quickly enough such that they have yet to be formally compared and evaluated to identify which ones are best for which situations (e.g., number of individuals in each “true” group, number of subgroups in a sample, unequal group sizes, small sample sizes) and what scientific questions. The Newman algorithm [19], as applied here, is widely used, but is likely not optimal for all situations. Importantly, many community detection approaches in the fMRI literature have been tested on relatively large adjacency matrices and, while useful, may not be optimal for smaller matrices where the nodes are participants.

Two, a few options exist regarding the generation of the similarity matrices (i.e., the adjacency matrix that indicates how similar each individual's connectivity weights are to each other individual) that were ultimately used with the community detection algorithm. For instance, the connectivity mapping results may vary based on preprocessing decisions, such as global signal regression (GSR). We used GSR because it offers a number of benefits such as improving the correspondence between resting-state correlations and anatomy [54] and motion correction at the time series level [55], [56]. There is the possibility that GSR altered connectivity patterns [57], [58], which in turn may change the degree of similarity of individuals and thus the resulting community designations. Hence future work can investigate the impact of these decisions on resulting subgroup designations.

Other options exist regarding the features of the connectivity maps used to generate the similarity matrix. We also could have created the similarity matrix using the estimates for lagged paths in addition to or instead of the contemporaneous path estimates. Since contemporaneous paths seem to hold information regarding neuronal relationships, we chose to use these. Lagged effects, and by extension, coherence in the frequency domain, could also be informative in creating groups. Future work is also needed to investigate approaches for arriving at the similarity matrix in a manner that utilizes the individual-level paths in addition to the sample-level ones. We utilize sample-level paths since they are normally distributed across individuals and thus appropriate for obtaining correlation values denoting similarity in values for use with the community detection algorithm. Additionally, the high degree of heterogeneity with regards to the individual-level paths necessitated that we omit these when creating the similarity matrices since including individual-level results via a distance measure (as opposed to correlation) resulted in groups that were too small to offer any value over individual-level analysis. As the solution to this problem is not straightforward, more work needs to be done to identify methods that arrive at similarity matrices that are appropriate for data that is not normally distributed.

Data of a substantial sample size are often capable of being split into a multitude of valid subgroup arrangements. What determines a given demarcation rests predominantly on the features (in our case connections) chosen to show similarities or differences between individuals and the algorithm chosen to demarcate the sample. What is most important in this line of work is to identify the subgroups that are most meaningful with regard to clinical translation. Thus, the work presented here will need to be validated further by examining how the subgroups predict outcomes and/or respond to treatment differently (as seen in [2]), and how different contextual factors interact with the brain physiology to predict diagnostic classification that can guide prevention efforts. The method presented here is a step towards this goal. Having demonstrated the feasibility and utility of dynamic community detection, more work is needed across multiple domains of inquiry to exploit the extent to which understanding heterogeneity in brain physiology can be helpful in guiding treatment, prevention, and intervention efforts.
